# CCR7 in Blood Cancers – Review of Its Pathophysiological Roles and the Potential as a Therapeutic Target

**DOI:** 10.3389/fonc.2021.736758

**Published:** 2021-10-29

**Authors:** Carlos Cuesta-Mateos, Fernando Terrón, Marco Herling

**Affiliations:** ^1^ Immunology Department, Hospital Universitario de La Princesa, Instituto de Investigación Sanitaria- Instituto la Princesa (IIS-IP), Madrid, Spain; ^2^ Immunological and Medicinal Products (IMMED S.L.), Madrid, Spain; ^3^ Catapult Therapeutics BV, Lelystad, Netherlands; ^4^ Clinic of Hematology and Cellular Therapy, University of Leipzig, Leipzig, Germany

**Keywords:** CCR7, lymph node, lymphoma, leukemia, immunotherapy

## Abstract

According to the classical paradigm, CCR7 is a homing chemokine receptor that grants normal lymphocytes access to secondary lymphoid tissues such as lymph nodes or spleen. As such, in most lymphoproliferative disorders, CCR7 expression correlates with nodal or spleen involvement. Nonetheless, recent evidence suggests that CCR7 is more than a facilitator of lymphatic spread of tumor cells. Here, we review published data to catalogue CCR7 expression across blood cancers and appraise which classical and novel roles are attributed to this receptor in the pathogenesis of specific hematologic neoplasms. We outline why novel therapeutic strategies targeting CCR7 might provide clinical benefits to patients with CCR7-positive hematopoietic tumors.

## 1 Introduction

Lymph nodes (LN) function as major immunological hubs, being essential for immune homeostasis and the generation of effective immune responses (1). LNs are also fundamental sites-of-origin in disease development and progression as well as in treatment failure of several hematological malignancies. Growing evidence suggests that cell trafficking orchestrated by the C-C chemokine receptor 7 (CCR7) plays a critical role in the pathophysiology of several leukemias and lymphomas. This receptor assists malignant cells in access to niches that provide proliferating cues and enable escape from therapy-induced death, hence, promoting disease progression and resistance. In this review we provide a summary of insights towards a better understanding in which blood cancers, particularly B-cell, T-cell, and myeloid-cell malignancies, CCR7 mediates which pathogenetic functions. We further appraise how this chemokine receptor is of great potential for the development of rational and effective therapies in some of these conditions.

## 2 CCR7: A Single Receptor Linking Innate and the Adaptive Immunity in the LN

The homeostatic chemokine receptor CCR7 (also known as Epstein–Barr virus-induced gene 1 (EBI1), Burkitt’s lymphoma receptor 2 (BLR2), or CD197) is a G-protein coupled receptor (GPCR) (2–4). CCR7 is expressed by various immune cells including double negative (DN) and single positive (SP) thymocytes, naïve, central memory, and regulatory T-cells (T_N_, T_CM_, T_REG_) as well as naïve B-cells, CD56^+^CD16^-^ regulatory NK cells, and semi-mature and fully mature dendritic cells (DC) (5–7). Generally, T-cell subsets and mature B-cells constitutively express CCR7 whereas NK cells and DC acquire CCR7 expression upon pathogen encounter (5). In homeostasis CCR7 specifically drives cell homing into LN and other secondary lymphoid organs (SLO) (8–10). This single GPCR orchestrates efficient interactions between different CCR7-expressing cell types that belong to both the innate and the adaptive functional axis of immunity and which immigrated to the LN from different peripheral environments. As part of this, CCR7 directs central aspects of immune cell migration into the LN: cell trafficking, firm arrest at the endothelium, extravasation, positioning within SLO, activation, differentiation, survival, and egress. All these processes mediated by CCR7 take place upon binding to either of its two cognate ligands, the chemokines CCL19 (also known as ELC or MIP-3β) and CCL21 (also known as SLC or 6CK), which are constitutively expressed by stroma cells in SLO and which are present on lymphatic vessels, high-endothelial venules (HEVs), and fibroblastic reticular cells (FBR) of the T-cell zones (5, 6, 11). Different signaling pathways downstream of CCR7 and several mechanisms differentially ignited by CCL19 or CCL21 signaling determine the overall outcome of CCR7 engagement in each cell type. A detailed review on those mechanisms is provided by Hauser et al. (11).

## 3 CCR7 Expression and Functions in Distinct Blood Cancers

Most of CCR7’s roles in homeostasis (e.g. cell trafficking, interstitial migration, or survival) are particularly relevant for leukemias and lymphomas, which very often express CCR7 because of their lymphoid origin and/or maturation stage. In this section we will appraise the current knowledge on CCR7 biology in several B-cell and T-cell cancers and in selected myeloid neoplasms. In addition, we will review the evidence that associates expression profiles of CCR7 with functionality and pathological findings such as LN infiltration or spread to the central nervous system (CNS).

### 3.1 B-Cell Malignancies

B-cell malignancies consist of distinct diseases that can arise throughout the developmental lifespan of a B-cell. From pro-B-cells in the bone marrow (BM), through circulating mature memory B-cells, each stage of B-cell development is prone to oncogenic alterations and transformation. The corresponding entities carry characteristic protein profiles, including differential expression of CCR7. In some diseases, expression can differ between malignant cells and the corresponding normal ontogenetic counterpart. In others, tumor-associated CCR7 expression can be unaltered, but may trigger different cellular functions.

#### 3.1.1 B-Cell Acute Lymphoblastic Leukemia (B-ALL)

B-ALL is the most common childhood malignancy and represents the leading cause of cancer-related deaths in children and young adults (12). B-ALL arises from a monoclonal or oligoclonal expansion of malignant B-cell precursors in the BM. Normally, CCR7 is not expressed by precursor B-cells (6, 13) and scant information is available on CCR7 expression and function in childhood and adult B-ALL. Indeed, reports are somehow controversial since gene expression profiles showed both unchanged (14, 15) or upregulated CCR7 mRNA (especially in pediatric B-ALL) (16, 17). Similarly, some protein studies performed in a low number of cases showed no CCR7 in B-ALL (18, 19) while, in others series, expression was detected in specific subtypes of B-ALL, mainly in pediatric Burkitt´s-like B-ALL and in one third of pediatric pro-B, early-pre-B, and pre-B ALL (20). In most cases, the surface levels of CCR7 tested by flow cytometry were low-to-moderate. In our hands, adult B-ALL showed detectable CCR7 in only a minor tumor cell fraction of 10-40% (13, 21).

Related to differential CCR7 functionality, isolated early pre-B-ALL cells showed spontaneous migration towards CCL19 (20) whereas normal pre-B and pro-B-cells showed chemotactic responses to this ligand only after a previous exposure to soluble recombinant CD40 ligand (CD40L). In fact, engagement of CD40 seems a common mechanism to up-regulate CCR7 in lymphoblastic cells from patients potentiating the migration towards CCL19 (22, 23). Interestingly, this phenomenon seems highly specific to CCR7 since pre-incubation with CD40L did not affect chemotaxis mediated by other chemokine receptors (e.g. CXCR4) (20). Nonetheless, robust expression data confirm that, in general, CCR7 is absent or found at variably low levels in B-ALL suggesting a rather low impact in mediating migration of this malignancy into LN or other lymphoid niches. This is in accordance with the low incidence of lymphadenopathy in B-ALL. However, CCR7 may provide competitive advantages to the minor fraction of leukemic cells that express this receptor, potentially enabling them to escape to non-lymphoid protective tissues. Indeed, a recent study on a cohort of 160 B-ALL could associate expression of CCR7 and of zeta-chain-associated protein kinase 70 (ZAP-70) protein with enhanced migration (24). These authors also showed that CCR7 expression at diagnosis correlated with cerebral manifestation, which led to the hypothesis that CCR7 is involved in preferred CNS homing in the first phases of the disease. Notably, similar mechanisms have been previously proposed for T-ALL (25). We will address the more established contribution of the CCR7/CCL19 axis in CNS infiltration and survival of T-ALL cells below.

Once at their protective niches, the minor fraction of homed CCR7-expressing B-ALL cells could utilize CCR7 also as a mediator of survival signals. In this context, synergisms between CCR7/CCL19 and CXCR5/CXCL13 were shown to mediate resistance of B-ALL cells to tumor necrosis factor alpha (TNF-α)-mediated apoptosis through activation of paternally expressed gene 10 (PEG10) (26, 27). Moreover, both ligands also synergistically regulated CD40-CD40L crosstalk between B-ALL cells and CD8^+^ T-cells leading to a PEG10-mediated enhanced production of IL-10 in CD40-activated leukemic cells, which impaired tumor-specific cytotoxic T-cell (CTL) responses (28). Similarly, CD40-acivated B-ALL cells can deplete IL-12 from the local milieu and block the differentiation process of CCR7-expressing naïve T-cells towards active T_H_1 effectors (22). Therefore, CCR7 is likely involved in the creation of tolerogenic niches and its expression might confer escape of B-ALL cells from immune surveillance.

#### 3.1.2 Chronic Lymphocytic Leukemia (CLL)

CLL is the most common leukemia in Western countries (29). It is characterized by the clonal proliferation and accumulation of mature, typically CD5-positive B-cells within the peripheral blood (PB), BM, LN, and spleen. Typically, blood circulating CLL cells are arrested in the G_0_/G_1_ cell-cycle phase, whereas leukemic cells within LN proliferate and receive protective anti-apoptotic signals (30, 31). CCR7 overexpression, as mRNA (14, 32–34) and as surface protein (in comparison to normal pan-B-cells and CD5^+^CD19^+^ B-cells), is consistently found in virtually all CLL, irrespective whether sampled from PB, BM, or LN (13, 19, 21, 35–48). In agreement, migration in response to CCR7 ligands is enhanced in CLL cells as compared to its normal cell counterparts (13, 35, 40, 46) and both expression and functionality have been associated with nodal disease involvement (13, 27, 35, 36, 38, 46, 47).

In CLL, genetic factors or polymorphisms involved in the overexpression of CCR7 remain uncovered but one single-nucleotide polymorphism (SNP) in the *CCR7* gene was strongly associated with the risk of acquiring CLL. Out of 6 tested SNPs (including rs11574665, rs2023906, rs2290065, rs3136685, rs3136687, and rs588019) (49, 50), the major G allele in the SNP rs3136687, which is located at the first intron and is in linkage disequilibrium with rs11574665, was associated with a higher risk towards CLL whereas the minor A allele resulted in a protective effect (49). The authors found no differences in CCR7 expression for such allelic variants. This lack of association between *CCR7* polymorphisms and receptor over-expression suggests that other proteins might ultimately determine different signaling pathways controlling *CCR7* gene transcription and/or surface protein expression. Accordingly, activating mutations at Notch1 intracellular domain were shown to repress the dual specificity protein phosphatase 22 (*DUSP22*) tumor suppressor gene that encodes a phosphatase that dephosphorylates STAT3 (51). Because of this, STAT3 is constitutively activated resulting in increased CCR7 levels in CLL cells. Another STAT family member, STAT-4, which is profoundly reduced in CLL cells (52), was implicated in *in vivo* down-regulation of CCR7 in T-cells (53). Different B-cell receptor (BCR) signaling pathways have been implicated as well in the aberrant up-regulation of CCR7. For instance, after BCR engagement, the ZAP-70 protein has been shown to up-regulate CCR7 through an extracellular signal-regulated kinase 1/2 (ERK1/2)-dependent mechanism (42). Similarly, the transcription factors NFATC1 (nuclear factor of activated T-cells), NF-κB (nuclear factor kappa B), and AP-1 (activator protein 1) are known to regulate CCR7 expression in CLL following activation *via* the BCR or other receptors (54–56).

In CLL, the normal LN architecture is effaced by the malignant infiltrate (57). Different studies confirm CCR7 as the main receptor involved in nodal entry of CLL cells. Mechanistically, binding to CCL21 on the surface of HEVs activates α4β1 (CD49d/CD29; VLA-4) and αLβ2 (CD11a/CD18; LFA-1) (35, 58), which respectively bind to ICAM-1 and VCAM-1. Whether β1 and β2 integrins are equally relevant in CCR7-mediated homing of CLL cells is still unknown. By one hand, Till et al. showed spontaneous active conformation (without chemokine-induced clustering) of LFA-1 on CLL cells (59). On the other hand, circulating CLL cells usually express low levels of these integrins (60). Therefore, few CLL cells are able to arrest in ICAM-1 expressing endothelium *in vitro* and to migrate to lymph nodes of NOD/SCID mice *in vivo* (60). However, a significantly higher expression of both types of integrins (thus facilitated access to LN) is detected in CLL cells derived from high-risk patients with unfavorable cytogenetic abnormalities such as deletion 17p, deletion 11q and, specially, with trisomy 12 (47, 61, 62). Recently, Legler et al. have shown on trisomy-12 carrying OSU-CLL cells that CCR7-mediated inside-out signaling to the β1 integrinVLA-4 and the β2 integrin LFA-1 is controlled by Src and ZAP-70 kinases (58, 63). This process is critical for static and dynamic cell adhesions to endothelium and subsequent migration, but did not seem to impact the speed of migration velocity, and was dispensable for chemokine-mediated crawling and diapedesis. Further studies are needed to know which are the molecular mechanisms driving the latter processes.

Activation of integrins promotes the production and release of MMP-9 (64) and the subsequent transmigration of CLL cells through the endothelial cell wall into the LN [transendothelial migration (TEM)]. This ability of CLL cells to eventually accumulate at these sites may be determined by the genetic background of CLL. The more aggressive *immunoglobulin heavy variable chain* (*IGHV)* gene unmutated CLL subset displays higher CCR7 expression (35, 36, 43, 46, 65). In support, the presence of adverse factors such as *IGHV* unmutated status or expression of CD38 or ZAP-70 was shown to be associated with increased responsiveness of CLL cells to CCR7 ligands in both chemotactic and TEM assays (38, 47, 60, 66, 67).

There is data that indicate CCR7 to drive interstitial migration within the lymphoid tissue and to facilitate the positioning of leukemic cells close to accessory CD40L^+^CD4^+^ T_H_ cells, follicular DC (FDC), and stromal cells (e.g. stromal-like cells and nurse-like cells), which are all known to promote the survival and growth of the malignant clone (9, 38, 68, 69). This crosstalk with accessory cells can induce the release of high levels of CCL19 and CCL21, which among others, has two functional consequences. First, it causes the establishment of a self-enhancing loop that recruits more CCR7-expressing tumor and accessory cells favoring the creation of a protective and tolerogenic microenvironment. Secondly, CCR7 ligands directly promote survival of CLL cells. In these scenarios CCL19 and CCL21 can act as single factors that activate mitogen-activated protein kinase (MAPK) and phosphatidylinositol-3-kinase (PI3K)-AKT signaling (40, 70) or in a cooperative fashion with CXCL13 which contributes to resistance of CLL cells (but not normal CD5^+^ B-cells) to TNF-α-mediated apoptosis through up-regulation of PEG10 which in turn stabilizes caspases-3 and -8 (26, 27).

Finally, besides its role in homing and survival within SLO, CCR7 along with sphingosine-1-phosphate receptor 1 (S1P1) are crucial molecules in the egress of lymphocytes from lymphoid organs to PB. As shown in CCR7-deficient mice, cells lacking CCR7 left LN quicker than wild-type cells. In contrast, overexpression of CCR7 retarded emigration from LN to PB (71). Evidence implicates the characteristic high CCR7 expression in CLL alongside the low expression of the egress receptor S1P1 to contribute to nodal retention (43, 46, 72), driving a scenario of lymphadenopathy that favors homing and accumulation in SLO. Remarkably, driving leukemic cells out of LN into PB induces death by deprivation of milieu-derived signals and is one of the modes of action (MOA) of a very efficient treatment option in CLL, ibrutinib, the first approved Bruton´s tyrosine kinase (BTK) inhibitor with activities on multiple other kinases (73). Recent work suggests ibrutinib to down-modulate CCR7 expression and function in CLL (e.g. integrin activation and receptor recycling) and by that to restore the balance between CCR7 and S1P1 and to enforce nodal egress of leukemic cells (44, 46).

#### 3.1.3 Mantle Cell Lymphoma (MCL)

MCL is a B-cell tumor that originates from the clonal expansion of a naïve CD5^+^ B-cell localized in the mantle cell zone surrounding the germinal center (GC) in secondary follicles of the LN (74). Lymphadenopathy and BM infiltration are the most common clinical manifestations, followed by splenomegaly and PB lymphocytosis. Gastrointestinal and CNS involvement have been reported as well (75–77). This preferential pattern of dissemination can be attributed to the high surface expression of CCR7 by MCL cells as per flow cytometry, second to those levels observed in CLL (13, 21, 78, 79). In most of these studies, MCL cells from LN, PB, BM, and pleural effusions expressed higher levels of CCR7 than their proposed normal counterparts. The underlying causes of this overexpression are largely unknown. Comparative transcript analysis between MCL and normal B-cells have shown CCR7 mRNA to be significantly up-regulated in lymphoma cells (32). The fact that CCR7 was not among the top differentially regulated RNAs in MCL (80) suggests that additional mechanisms such as altered protein turn-over (46) are responsible for overexpression of surface CCR7 in MCL. Nevertheless, MCL and normal B-cells differ in their migratory behavior towards CCR7 ligands. In chemotaxis assays, MCL cells, but not their normal counterparts, migrated in response to CCL19, which was selectively potentiated by pre-exposure to CXCL12 (78). These results suggested CCR7-driven migration to be of relevance in the dissemination pattern seen in MCL patients. We corroborated this hypothesis in pre-clinical *in vivo* models in which the inhibition of CCR7 by anti-CCR7 antibodies abrogated infiltration of CCR7-expressing MCL cell lines into LN, spleen, lung, or CNS, all of them tissues in which CCR7 ligands are found (21). Moreover, this neutralization of the CCR7 axis also induced a strong reduction in viability of lymphoma cells within tumor masses, confirming that in MCL CCR7 overexpression is not only involved in orchestrating migration, but also in directly promotion of survival.

#### 3.1.4 Follicular Lymphoma (FL)

FL is the second most common type of non-Hodgkin´s lymphoma (NHL) and despite its indolent nature, it is essentially incurable (81). FL encompasses lymphomas emerging from a GC B-cell, which can vary in presentation from indolent to aggressive courses (82). Similar to normal GC lymphocytes, which physiologically down-regulate CCR7 and up-regulate CXCR5 (83), FL cells express low to moderate levels of CCR7. Moreover, the proportions of CCR7-expressing cells were reported to be low and, in some patients, no expression of CCR7 was detected at all (13, 19, 21, 79). In agreement, mRNA levels in FL cells did not differ from those of their normal counterparts (32, 84–87). The genetic variants of CCR7 rs2023906, rs2290065, rs3136685, and rs588019, were not associated with differential expression or with clinical course in FL (50). The fact that CCR7 is not prominently found in most FL suggests that it has a limited role in the pathophysiology of this lymphoma, which is supported by recent evidence. In fact, comparative analyses of LN from FL *versus* reactive LN revealed that CCL21 and CXCL12 were neither over- nor differentially expressed, whereas FL-LN nearly lacked expression of CCL19. In addition, in FL lymphoid tissues in which both CCR7 ligands were detected, they were preferentially found in HEVs and in lymphatic vessels of T-cell zones, but on average at 3-fold lower levels than in reactive LN (88). Conceivably, the reduced abundance of CCL19/CCL21 in LN of FL is lymphoma instructed and contributes to evasion from anti-tumor immunity. Accordingly, FL progression may be associated with reduced numbers of perifollicular CCR7^+^ gamma-delta T-lymphocytes due to a shortage of attracting CCR7 ligands (88).

#### 3.1.5 Burkitt`s Lymphoma (BL)

CCR7 was first characterized in EBV infected BL cell lines, hence the initially coined term Epstein-Barr-induced 1 (EBI-1) for CCR7 (4). CCR7 upregulation was shown to rely on the viral transactivator EBV nuclear antigen 2 (EBNA-2), which after binding to centromere-binding factor 1 (CBF-1, also known as RBP-jκ), a highly conserved cellular DNA binding repressor, gains access to regulatory regions of CCR7 target genes and activates transcription in infected (previously EBV‐negative) BL4 BL cells (4, 89). Information on CCR7 expression in primary BL material is scarce. No upregulated mRNA levels were seen in 22 patient samples (32, 84) and, to our knowledge, only one work studied CCR7 expression by flow cytometry in another 9 patients (79). The receptor was found in all cases, but in a fraction of ~53% of tumor cells per sample with no disclosed results on receptor surface levels. Interestingly, in the NC37 BL cell line, *in vitro* chemotaxis and TEM was modulated by a cooperative activity of CXCL12 with CCL19 or CCL21, suggesting that the CCR7 axis is involved in BL cell homing to LN (90). Accordingly, in the syngeneic *Eμ-Myc* mouse model of BL, CCR7 was found necessary for lymphoma cells to home to LN (9). These results also indicated that CCR7 guides tumor cells to distinct microanatomic sites in spleen and LN, especially to their T-cell zones. Cross-talk with resident stromal and accessory cells at these sites contributed to the creation and preservation of pro-tumor niches that conferred a survival advantage to CCR7-positive lymphoma cells over CCR7-deficient lymphoma cells (9). In a proposed model, stromal cells (e.g. fibroblastic reticular cells, FRC, and HEV endothelial cells) secrete CCL21 through which CCR7-expressing lymphoma cells home through HEV into the LN (or spleen) and migrate towards FRC in the T-cell region. Upon interaction with FRC, lymphoma cells secrete lymphotoxin through which they stimulate lymphotoxin-β-receptor expressing FRC. In turn, BL cells receive survival signals, presumably including Indian hedgehog (Ihh) secreted by FRC and CD40 stimulation through CD40 ligand-expressing CD4^+^ T-cells located in the T-zones. The importance of this cross-talk was demonstrated by showing that genetic deletion of CCR7 impaired lymphoma growth (9). Therefore, this model not only established the basis for a better understanding of the pathogenic role of CCR7 in BL, but also in many other blood cancers with a high dependence on the nodal or splenic microenvironments.

#### 3.1.6 Subsets of Diffuse Large B-Cell Lymphoma (DLBCL)

DLBCL, the most common type of malignant lymphoma, accounts for ~30% of adult NHL (91). DLBCL can arise at multiple anatomical sites and comprises two major groups: activated B-cell like (ABC) and GC B-cell like (GCB) DLBCL. Therefore, it is not surprising that chemokine receptor (CKR) expression varies between these subtypes and in association with disease location (91, 92). Up to 62% of DLBCL express CCR7, both in analyses of flow cytometry and immunohistochemistry (IHC), with a preferential mRNA and protein expression in the non-GCB subtypes, especially in patients with both LN and BM involvement (79, 85, 93, 94). In EBV-positive DLBCL of the elderly, in primary effusion lymphoma, in gastric extranodal DLBCL, and in transformation of gastric mucosa-associated lymphoid tissue (MALT) lymphomas to gastric extranodal DLBCL, up-regulation of CCR7 mRNA, among other CKR, was reported (32, 91, 94–96). Notably, in EBV-associated DLBCL recurrent mutations in the *CCR7* gene are found in 11% of patients (94). These alterations seem exclusive to this subtype and could enable homing of tumor cells to SLOs where the virus in turn propagates infection or establishes latency, thereby driving lymphomagenesis (97). In other related primary lymphomas such as intravascular large B-cell lymphoma and mediastinal large B-cell lymphoma a characteristic decrease in immunodetected CCR7 was described (32, 98, 99). As these types typically show sparing of LN manifestation, this corroborates the role of CCR7 in nodal homing. The genetic polymorphisms in *CCR7* that were disclosed in FL did not associate with the risk of acquiring DLBCL (50). CCR7 expression, both at mRNA and protein levels, was an independent prognostic factor for disease progression, advanced clinical stages, shorter median survival times, and poorer survival rates in GC and ABC DLBCL (93, 100). First functional data on CCR7 in DLBCL indicate that receptor expression facilitates CCR7‐mediated *in vitro* migration in EBV‐positive DLBCL cell lines with functional analyses on primary samples still missing (91).

#### 3.1.7 Primary Central Nervous System Lymphomas (PCNSL)

Immunohistochemical staining of PCNSL and secondary CNS lymphoma (sCNSL) showed these disorders to present CKR profiles that were different from those of systemic DLBCL. CCR7 was detected in the malignant B-cells of specimens of PCNSL (101) and in CNS relapses of DLBCL (102). However, and opposed to lymphomas with peripheral involvement, CCR7 was present in the cytoplasm rather than at the cell surface indicating that the receptor may not respond to its corresponding ligands in the same conventional fashion (101). It is tempting to associate this loss of surface CCR7 with the absence of nodal involvement, which defines PCNSL (103). However, one should also take into account that the restricted intracellular CCR7 expression pattern may in part be a consequence of a milieu that is highly enriched in CCR7 ligands, especially in CCL19, the most potent inducer of CCR7 endocytosis (11). In agreement, a recent study addressing the role of gliosis in lymphoma cell retention in the CNS found that astrocyte-derived CCL19 was required for gliosis-promoted CNSL *via* enhancing parenchymal retention of lymphoma cells (104).

#### 3.1.8 Marginal Zone Lymphoma (MZL)

MZL comprises three entities that arise from the marginal zone surrounding the follicular GC of the LN: extranodal MZL or MALT lymphoma, splenic MZL, and nodal MZL. Analyses on MALT lymphoma samples showed more than 50% of malignant cells to express CCR7 (79). In extragastric MALT lymphoma or malignant transformation from *Helycobacter pylori*-associated gastritis to MALT lymphoma, up-regulation of CCR7 mRNA, among other CKRs, was a common finding (91, 95, 96). In splenic MZL, no changes were seen at the mRNA level (87) whereas flow cytometry revealed significantly reduced expression of CCR7 as compared to normal B-cells (13, 19, 21, 79). The low CCR7 expression in extranodal or splenic MZL suggest a minor role of this receptor in their pathobiology. This in turn might explain the minimal lymphadenopathy seen in these types of MZL (13) and reports on CCR7 expression in nodal MZL are still missing. Accordingly, one study in salivary gland MALT lymphoma samples selectively implicated the chemokine CCL21 in the organization of ectopic reactive lymphoid tissue whereas no significant expression of the ligand was detected in the malignant lymphoid aggregate (105). The authors concluded that CCR7 plays no major role in the infiltration of the epithelium or in the regulation of malignant cell survival.

#### 3.1.9 Hairy Cell Leukemia (HCL)

HCL is an indolent, rare disease that accounts for approximately 2% of leukemias and is typically defined by the B-raf kinase mutation pV600E (106). Cell surface expression of CCR7 is low (or absent) in HCL cells when compared to normal B-cells (13, 19, 21, 107). Similarly, CCR7 transcription in HCL samples is reduced (32). This would explain why nodal dissemination is not a key feature in this disease.

#### 3.1.10 Lymphoplasmacytic Lymphoma (LPL) and Multiple Myeloma (MM)

LPL and its subgroup Waldenstroem´s macroglobulinemia (WM) are rare and indolent lymphomas that arise from terminally differentiated B-cells that physiologically do not express CCR7 (82). Analyses on very few clinical samples did not shed light on the expression profile of CCR7 in LPL, as some cases did express the receptor (21) while others did not (13). Available evidence does not link CCR7 to an altered genetic profile in plasma cell leukemia (PCL) (108). Normal plasma cells and those of MM do not typically express surface CCR7, and if expressed, it is found in a minor proportion of cells as demonstrated in samples from BM or extramedullary sites (PB and pleural effusions) (13, 21, 109, 110). Curiously, in non-Hispanic Caucasian subjects the genetic CCR7 variant s3136685 was reported to be associated with an elevated risk for MM (110). Since this genotype was not associated with MM in further permutation-based tests and since previous evidence did not link CCR7 to MM (108, 111), these findings should be interpreted with caution and further investigation is required to clarify this issue. Finally, gene expression profiles of monoclonal gammopathy of undetermined significance (MGUS) and of smoldering myeloma seem to discard a prominent role of CCR7 in these conditions as well (108, 111, 112).

#### 3.1.11 Hodgkin Lymphoma (HL)

HL is a unique type of B-cell lymphoma characterized by the presence of a minority (<1%) of neoplastic cells in a background of infiltrating reactive cells (113). The microenvironment is considered to be shaped by the malignant cells and provides survival signals and protection against anti-tumor immune responses (114). Based on differences in histopathology, HL is classified in two subgroups: the classical form (cHL) that accounts for 95% of all HL cases and the nodular lymphocyte predominant variant (NLPHL) that represents only 5% of all cases (113). Tumor cells in cHL are termed Reed/Sternberg (RS) cells, which generally express CD30, whereas tumor cells in NLPHL are called lymphocytic and histiocytic (L&H) cells and lack CD30.

CCR7 expression has been observed in cHL-derived tumor cell lines and in primary tissue. In the majority of cell lines expression was moderate-high and CCR7 was functional in inducing migration towards both of its ligands (115). In patient samples, IHC revealed a differential expression of CCR7 between cHL and NLPHL. The classical form, located in the interfollicular zones, showed strong CCR7 expression whereas NLPHL, regularly associated to follicles, was shown to be CCR7 negative (115). Accordingly, mRNA levels were highly expressed in cHL when compared to NLPHL and normal B-cells (84, 116). Moreover, CCL19/CCL21 were found in tumor infiltrates of cHL, whereas the tumor nodules in NLPHD almost completely lacked these chemokines (115).

In cHL, CCR7 upregulation might be a consequence of two, or more, altered pathways that are partially interconnected. For example, the *CCR7* gene contains binding sites in its promoter region for the transcription factors AP‐1 and NF‐κB (3), and both axes have been shown to be constitutively active in cHL and to upregulate CCR7, individually or cooperatively (3, 117). Notably, the combined constitutive activation of AP‐1 and NF‐κB mimics a state of chronic inflammation that involves the production of cytokines by RS cells. Whether CCR7 up-regulation is part of the prominent NF‐κB program in cHL, as it is the case for CD30 expression (115, 118), remains to be investigated.

At the functional level, constitutively active WNT signaling in cHL is important in CCR7-mediated migration and generation of protumorigeneic milieus. Binding of the WNT protein to the low-density lipoprotein receptor-related protein 5/6 (LRP5/6) regulates CCL19-guided chemotaxis through the β-catenin and lymphocyte enhancer-binding factor-1 (LEF-1) pathways (119). WNT signaling is commonly involved in metastasis and angiogenesis in various tumors (120). In tumor cells of cHL canonical WNT/β-catenin/LEF-1 signaling is also required to secrete vascular endothelial growth factor A (VEGF-A), and by that, to attract endothelial cells as well as to enhance their migration, sprouting and tube formation. Therefore, canonical WNT signaling is a regulator of the endothelium-lymphoma interplay. WNT is a prerequisite for secretion of VGEF-A by cHL cells which stimulates biogenesis of vascular endothelium which in turn presents CCR7 ligands that direct movement of cHL cells towards vascular niches. Thus homing and interstitial movement of tumor cells within the affected LN is facilitated by constitutive WNT. Moreover, CCR7’s ligand, CCL21, was shown to be absent on RS cells, but was detected on the majority of small vessels (including HEV) with a luminal membranous localization (121).

The CCR7 axis not only seems to play a pathogenic role by recruiting cHL tumor cells, it is also implicated in recruiting pro-tumorigenic CCR7-expressing immune cells from the circulation. Within infiltrating immune cells in cHL, CCR7 (and the related homing markers CD62 and lymphocyte function-associated antigen 1, LFA1) were demonstrated to be expressed on a large proportion (~33%) of reactive T-cells, which showed receptor-mediated chemotaxis that was similar to PBMC from healthy donors (121). Notably, in cHL the infiltrate is commonly enriched by CCR7^+^ T_REG_ and activated T-cells (122–126). In contrast, in NLPHL these T-cell subsets are less abundant and are found outside the tumor area (115, 127). Together, these findings suggest different immune escape mechanisms in both subtypes of HL that may be related to the different expression profiles of CCR7 in tumor-associated cells and of CCR7 ligands in the surrounding tissue.

### 3.2 CCR7 in T-Cell Malignancies

As described for B-cell malignancies, T-cell neoplasms consist of multiple entities that are thought to arise from particular stages of T-cell development. For instance, T-cell acute lymphoblastic leukemia (T-ALL) originates from thymic stages of T-cell evolution while peripheral (post-thymic) T-cell neoplasms show features of mature T-cells with distinct phenotypes of differentiation, e.g. T-cell prolymphocytic leukemia (T-PLL) mostly resembling T_CM_ or unconventional transitional stages between T_N_ and T_CM_; adult T-cell leukemia/lymphoma (ATLL) resembling T_REG_; Mycosis fungoides (MF) resembling T_EM_; Sézary syndrome (SS) resembling T_CM_; or T-cell large granular lymphocytic leukemia (T-LGL) resembling activated cytotoxic T-cells (113, 128–130). Based on this phenotypic characterization, CCR7 expression would follow its physiological T-lineage pattern and be highest in those diseases resembling a DN or SP thymocyte, T_N_, T_REG_, or T_CM_ and to show LN or CNS involvement. A low number of studies limits the knowledge on the role of CCR7 in some of these disorders. However, in light of the restricted armamentarium of available efficient therapies for T-cell malignancies, such insights are highly desired. In this section we will address our current knowledge on CCR7 biology in several T-cell cancers and will try to associate reported expression profiles with CCR7 functionality and pathological findings.

#### 3.2.1 T-Cell Acute Lymphoblastic Leukemia (T-ALL)

T-ALL mainly afflicts children and adolescents. It presents with increased white blood cell counts and often with hepatosplenomegaly. At relapse, there is an increased incidence of CNS manifestations (131, 132). A seminal report showed CCR7 to be a functional receptor that is highly expressed in 4 of 5 T-ALL cell lines and in PB tumor cells of 8 of 11 T-ALL patients (25). A recent study in a larger cohort of 130 patients (24) and unpublished data from our laboratory confirm these results. In T-ALL, CCR7 expression is controlled by the activity of the oncogene *Notch1*. Significantly up-regulated CCR7 was found in human T-ALL cells that harbor *Notch1*-activating mutations while receptor expression was repressed by Notch1-specific γ-secretase inhibitors (DBZ or compound E), both at mRNA and at protein levels (25). Mechanistically, Notch receptor engagement initiated the PI3K/mammalian target of rapamycin complex 2 (mTORC2) pathway, which transmitted through NF-κB to regulate expression of the *CCR7* gene in leukemic cells (25, 133). Notably, in pre-clinical *in vivo* T-ALL models generated by overexpression of the intracellular cleaved form of Notch1 (ICN1), CCR7 overexpression led to enhanced chemotaxis and invasion into different tissues, especially to leptomeningeal spaces of brain and spinal cord, in which endothelial cells were shown to produce CCL19 (25). This CCR7-driven homing into CNS facilitated leukemic cell survival and was associated with reduced animal survival. Similarly, CCL19 promoted T-ALL cell invasion of spleen in syngeneic *in vivo* models and shortened host survival (134). Inside cerebral or spleen parenchyma, cross-talk between stromal cells and leukemic cells mediates the production of higher levels of tissue CCL19 (25, 134, 135). In CNS, these positive loops and the concomitant alteration of drainage from cerebrospinal fluid facilitated lymphoblastic meningeal infiltration (25). Nonetheless, it is likely that CCR7 is not the sole mediator of this process as meningeal infiltration is also detected in ICN1-induced T-ALL with CCR7-deficient hematopoietic progenitors (135). A recent study suggested that CNS infiltration in xenograft models is regulated by ZAP-70 which positively correlated with the overexpression of both CCR7 and CXCR4 and with migratory abilities towards CCL19 and CXCL12 (24). This study also confirmed, in a large cohort of 130 T-ALL patients, the positive correlation between ZAP-70 and CCR7 expression and, importantly, high CCR7 expression in tumor cells from BM biopsies at diagnosis was associated with a significant 11-fold increased risk of CNS involvement (24). Together, despite some T-ALL patients showing low or absent expression of CCR7 in their tumor cells from BM (135), the herein presented evidence supports CCR7 as a key element responsible of high-risk features such as CNS infiltration.

#### 3.2.2 T-Cell Prolymphocytic Leukemia (T-PLL)

Although being the most frequent mature T-cell leukemia in Western countries, T-PLL represents only ~3% of all T-cell malignancies (136–138). Its clinical course is typically aggressive with poor responses to conventional chemotherapies resulting in a median overall survival (OS) of usually <2-3 years (139, 140). An inevitably rapid proliferation of mostly CD4^+^ prolymphocytes involves the PB, BM, spleen, liver, LN as well as skin and effusions (136, 137, 141). Not uncommon are CNS involvements (136, 137, 142). This pattern of dissemination suggests chemokine receptors to play an important role in T-PLL, however, little is known about their relevance and the role of their ligands in the pathophysiology of T-PLL (143). Although previous evidence did not show overexpression of CCR7 mRNA in six primary T-PLL samples (144), a recent study by our groups focused on CCR7 in T-PLL biology and its interventional potential (130). We assayed CCR7 surface levels at diagnosis by flow cytometry in 109 patients and found that receptor overexpression in malignant cells is seen in a very high proportion of cases (86.5%). CCR7 expression profiles were also instrumental in assigning T-PLL to stages of memory T-cell differentiation (130, 145). The proportion of CCR7-expressing T-PLL cells in PB at diagnosis was associated with a shorter OS and a higher risk of death within an 8-year follow-up period (130). CCR7 was a fully functional receptor upon CCL19 and CCL21 binding and its downstream signaling pathways activated PI3K and ERK (130), two axes that have shown to be relevant in T-PLL pathogenesis (145–147). We further showed that receptor activation triggered chemotaxis, invasion trough biological matrices or endothelial cells, and T-PLL cell survival (130). In *in vivo* pre-clinical studies, we confirmed CCR7 to play critical roles in enabling tumor cells to access tumor microenvironments in CNS and lymphoid organs, especially in LN (130). In agreement, prominent HEV are often infiltrated by neoplastic cells in T-PLL (148), which suggests CCL21 as a major route for homing into lymphoid tissues and in mediating the dissemination of T-PLL cells to different organs. Our results demonstrated CCR7 to promote a rapid niche colonization as well as survival and proliferation in these environments.

#### 3.2.3 Adult T-Cell Leukemia/Lymphoma (ATLL)

ATLL is an aggressive peripheral T-cell malignancy associated with human T-cell leukemia virus, type 1 (HTLV-1) infection and predominantly occurs in HTLV-1 endemic areas such as South-Western Japan, the Caribbean Islands, Central and South America, intertropical Africa, and the Middle East (149, 150). The prognosis of ATLL is very poor with a 4-year OS rate of 11%, 16%, 36%, and 52%, in the subtypes of acute, lymphoma, chronic, and smoldering ATLL, respectively (151, 152). In the majority of cases, ATLL cells express CD4 and CD25 and often lack CD7 (152–154). Forkhead box P3 (FoxP3) expression is detected and led to concepts of ATLL cells to resemble T_REG_ (155) which, however, remains a subject of debate (156). The malignant cells of ATLL express surface CCR7 (157) and up-regulated CCR7 transcripts are associated with the aggressive acute ATLL subset, which distinguishes these cases from the less aggressive chronic ATLL (158, 159). Studies in larger cohorts confirmed upregulated CCR7 mRNA and protein, especially in patients with acute, progressive, or treatment refractory acute disease. These reports also associated higher CCR7 expression levels with a poor prognosis and nodal involvement (154, 160, 161). Accordingly, ATLL cells from patients with lymphadenopathy and splenomegaly showed enhanced ability to adhere to surfaces coated with intercellular adhesion molecule 1 (ICAM-1) and to migrate towards CCL19 or CCL21 (157). Recent whole-exome sequencing studies revealed gain-of-function mutations in the receptor (162, 163). The *CCR7* gene was recurrently and significantly affected in 11% of ATLL with a majority of cases harboring mutations that led to truncated protein forms at the C-terminal cytoplasmic domain, which regulates multiple biological processes. Of special interest were the mutations at CCR7 Trp355, which prevented receptor turn-over and internalization upon ligand stimulation resulting in increased surface receptor expression. These mutations led to an enhanced ligand-induced chemotaxis and PI3K/AKT signaling (162, 163). More recently, *CCR7* gene mutations were mutually associated with mutations at *phospholipase C gamma 1* (*PLCG1*) and *caspase recruitment domain family member 11* (*CARD11*) genes, which are frequent alterations in TCR/NF-ĸB signaling (164). The pathological implications of this coexistence in ATLL remain unaddressed.

#### 3.2.4 Mycosis Fungoides (MF)

MF is the most common type of cutaneous T-cell lymphoma (CTCL), in which a protracted clonal expansion of atypical dermatotropic CD3^+^CD4^+^ T-lymphocytes underlies a chronic cutaneous manifestation (165). The majority of patients with early-stage (i.e. limited patch/plaque) disease have a normal life expectancy, while in advanced (i.e. ubiquitous, tumor, nodal) stages survival is drastically reduced, which in addition to a marked symptomatology requires multimodal treatments (166–168). Available data emphasize a complementary, prominent pro-tumorigenic role of distinct factors present within the skin or LN milieus of CTCL, such as chemokines (CCL21 or CXCL12) (169), cytokines (IL-13) (170) or antigens able to entertain chronic T-cell receptor stimulation (171).

Expression of CCR7 has been considered a marker of advanced MF and a component involved in the spread of cutaneous lesions to lymphoid tissues. Indeed, single-cell RNA sequencing of skin biopsies from one patient with aggressive disease showed that malignant clones in PB and LN displayed a transcriptional program reminiscent of a more central CCR7^+^ memory-like phenotype, while retaining tissue-homing receptors (i.e. CLA, CCR10) (172). Nonetheless, evidence of CCR7 protein expression in MF samples is scarce. Kallinich et al. analyzed expression of several CKRs in MF (165). They studied CCR7 expression in skin biopsies from six patients with early disease and six patients at the tumor stage. Using IHC, they found no expression of CCR7 in any tested sample, however, two skin samples of advanced disease showed strong and uniform expression of CCR7 on tumor cells by flow cytometry. A more recent study reported CCR7 expression in 62% (13/21) of specimens as per IHC, and indicated that CCR7 expression strongly correlated with subcutaneous extension of lymphoma cells (173). The CCR7-expressing MF cell line MyLa shows enhanced *in vitro* migration towards CCL21 in an mTOR-dependent manner (161) and through up-regulation of *metastasis-associated lung adenocarcinoma transcript 1* (*MALAT1*) (174), a long noncoding RNA that is also associated with migration of several solid tumor types (175). These authors also concluded that CCR7 promotes subcutaneous involvement of MF. In agreement, total RNAs from skin biopsies of epidermis *versus* those from involved dermis of MF associated the presence of tumor cells in the dermis with the CCR7/CCL21 axis (161). Accompanying IHC analyses confirmed that expression of CCR7 was high in infiltrating lymphoma cells. It also demonstrated CCL21 in the cytoplasm of epidermal keratinocytes and to be diffusely distributed in the dermal extracellular matrix.

#### 3.2.5 Sézary Syndrome (SS)

SS is a mature systemic T-cell malignancy in which skin-homing T-lymphocytes also accumulate in PB and LN. Patients are highly symptomatic (e.g. pruritus, staphylococcal infections) and prognosis is poor with a median of survival of 63 months (166, 167). Despite increasingly better knowledge on disease biology, currently applied therapies show short-lived responses (168). By convention, SS has been regarded as a systemic variant of MF based on identical cytologic and immunophenotypic features. In addition, long-standing MF may subsequently develop into a secondary SS-like disease that exhibits circulating neoplastic cells, indistinguishable from those of primary de-novo SS. Nonetheless, several studies provide clues to consider SS and MF as two separate entities. First, patients with primary SS typically experience a more aggressive disease course, characterized by frequent involvement of LN (166–168). Second, MF and SS tumor cells show different molecular and CKR profiles (176–178). SS is thought to arise from expansions of mature long-lived CD4^+^CD7^-^ T-cells with a CD45RO^+^CD27^+^CD62L^+^ T_CM_ phenotype accompanied by a consistently high CCR7 mRNA and protein expression, whereas CCR7 expression in the predominantly T_EM_ cells from cutaneous MF lesions is controversial (165, 178–182). Admittedly, biases by the different sources of sampling, e.g. skin preferentially for MF *versus* blood and LN for SS might attribute to the observed differences.

Although CCR7 gene expression could not be significantly correlated with lymphoid organ involvement or patient survival in SS (179), it appears plausible that production of CCL19 and CCL21 by stromal and endothelial cells in lymphoid tissues contributes to the lymphotropism of SS cells. In support, the chemokine CXCL13, mainly produced in lymphoid tissues, promotes a synergistic CCR7-mediated migration that was of higher efficiency in SS cells than in normal T-cells (183). Additionally, CCR7 activation enhanced invasion by modulating adhesion and secretion of metalloproteases in clinical samples and in SS cell lines (183, 184). CCR7-induced integrin activation and metalloprotease secretion are processes that in other CCR7-expressing blood cancers are known to be required for CCR7-mediated TEM and for homing (35, 130).

Epidermotropism and tumor growth within the skin environment of SS are features that had been attributed to CCR7 function, but the exact mechanisms are poorly characterized. A first report found no function for CCR7 in promoting *in vitro* survival or proliferation of primary SS tumor cells (183). Another study on a cohort of 43 SS cases found contradictory results (169). This more recent investigation demonstrated that overexpressed CCL21 (and CXCL12) in skin tissue induced activation of PI3K/AKT/mTORC1 signaling in skin-resident SS cells. SS samples frequently show a recurrent loss of the phosphatase PTEN (phosphatase and tensin homolog) and the liver kinase B1 (LKB1) (169), two proteins that under normal conditions attenuate upstream activation of mTORC1 in low energy conditions (185). Therefore, these defects might result in a constitutive TORC1 activation that promotes protein translation and a metabolic shift from oxidative phosphorylation (mainly observed in quiescent/memory lymphocytes) toward aerobic glycolysis (typically observed in activated lymphocytes) (185). This increase in glucose demand (also known as Warburg effect) might be energetically beneficial during the recruitment of SS cells to skin and/or LN by CCL21, with the latter being able to further enhance mTORC1 activation and by that SS cell growth (169). Indeed, among other, in part stronger stimuli such as IL-2/IL-7, CCL21-mTORC1 also promoted up-regulation of the Ki67 proliferative protein in SS-derived cell lines and in primary SS cells (169).

#### 3.2.6 T-Cell Large Granular Lymphocytic Leukemia (T-LGL)

T-LGL is characterized by the chronic low-level expansion of mostly CD8^+^ T-cells in blood, BM, and spleen. Nodal disease is infrequent. T-LGL cells express pan-T-antigens, programmed cell death 1 (PD-1), some NK-cell associated molecules, cytotoxic granules (containing perforin and granzymes) and lack the CD28 co-stimulatory receptor. Detection of CD45RA and further markers of T-cell differentiation suggest a terminally differentiated effector memory (T_EM-RA_) phenotype (128, 186–188). T_EM-RA_ cells are featured by the absence of CCR7, and accordingly, most T-LGL cases in these studies did not show tumor cell expression of CCR7.

#### 3.2.7 Other T-Cell Malignancies

In other types of T-cell neoplasms such as peripheral T-cell lymphoma not otherwise specified (PTCL-NOS), extra-nodal NK/T-cell lymphoma (ENKTL), anaplastic large cell lymphoma (ALCL), and angioimmunoblastic T-cell lymphoma (AITL) expression of CCR7 remains poorly studied and controversial (including the source of expression, namely tumor cells *versus* local bystander cells). Two studies in a total of 41 ALCL patient samples and in 7 ALCL cell lines found the anaplastic-lymphoma kinase (ALK)-negative ALCL variant to overexpress CCR7 genes (compared to ALK-positive or primary cutaneous ALCL) (116, 189) while another series on LN biopsies associated ALCL to a CD4^+^CD45RO^+^CD27^-^ T_EM_ phenotype that lacks CCR7 (190). In contrast, PTCL-NOS showed CCR7 expression as part of its T_CM_ signature (190). However, gene expression profiles showed no upregulation of CCR7 transcripts in PTCL-NOS as compared to normal T-cells; as also observed for AITL (191). A subsequent study reported expression of CCR7 by IHC in an overall of 83% of samples that contained PTCL-NOS, ENKTL, ALCL, and AILT, but without disclosed resolution for the proportions of CCR7 positive cases per entity (184). Despite this shortcoming, this work corroborated the expression of CCR7 in a high proportion of mature T-cell malignancies and, importantly, significantly associated CCR7 staining with lymphatic or hematogeneous dissemination as well as with clinical stage. As in B-cell malignancies, constitutive activation of the transcription factor AP-1 is a proposed mechanism underlying CCR7 overexpression and CCR7-mediated cell survival in some of these conditions, particularly ALCL (117).

In other, very rare T-cell lymphomas, studies on CCR7 have been sporadically reported. In a case of primary cutaneous aggressive epidermotropic CD8+ T-cell lymphoma transformation from an indolent to an aggressive phase was accompanied by a shift to CCR7 expression (177). A case of enteropathy-associated T-cell lymphoma (EATL) showed no lymphoma-cell associated CCR7 (178), fitting its cytotoxic T-cell nature, similar to CCR7-negative T-LGL.

#### 3.2.8 Natural Killer (NK) Cell-Type Lymphoproliferative Diseases

NK-cell cancers can be subdivided into aggressive NK cell leukemia (ANKL) and indolent chronic NK cell lymphocytosis (CNKL), both characterized by leukemic infiltration into multiple organs (192). In a cohort composed of PB samples of nine ANKL and six CNKL cases several CKR were investigated by flow cytometry (193). In both types of leukemia, CCR7 was detected in a small proportion of tumorous NK-cells (<25%), a lower proportion than the relative number of CCR7-positive NK-cells the authors found in six healthy controls. Together, these results suggested that CCR7 might not play an important role in the pathophysiology of ANKL or CNKL.

### 3.3 CCR7 in Myeloid-Cell Malignancies

Description of CCR7 in myeloid-cell derived cancers is anecdotal and, as opposed to lymphoid disorders, myeloid neoplasms seem to be mainly characterized by downregulated CCR7, although this aspect still remains controversial.

#### 3.3.1 Myelodysplastic Syndrome (MDS)

MDS constitutes a heterogeneous group of clonal hematopoietic stem cell diseases that share ineffective hematopoiesis, increased risk of developing acute myeloid leukemia (AML), and augmented prevalence of immune deregulation. To our knowledge no studies have addressed the expression or functions of CCR7 in myeloid cells from MDS patients. A comparative study of a cohort of 33 MDS, a condition with a known prominent inflammasome, patients with healthy controls found that in MDS CD8+ T-cells exhibited decreased levels of CCR7 and a concomitant upregulation of CCR3, CCR5, or CX3CR1 (194). Hence, a central pathogenic relevance of CCR7 and other CKR in MDS still has to be shown.

#### 3.3.2 Acute Myeloid Leukemia (AML)

AML is a heterogeneous group of aggressive proliferations with variable genetic make-up and differential responses to therapy (195). In clinical practice, CCR7 is sporadically detected by flow cytometry in AML samples from PB and BM in a small proportion of tumor cells (unpublished data). In agreement, CCR7 mRNA is not highly abundant in AML in several transcriptome analyses (14, 16, 196, 197). Only one study reported CCR7 transcript over-expression (~3-fold) in 148 human AML samples as compared to 12 samples of normal cord blood-derived CD34+CD45RA- cells (198). Protein expression to confirm high membrane levels of CCR7 was not studied. Potential associations of CCR7 mRNA with the most frequent genetic aberrations were also not investigated (198).

#### 3.3.3 Blastic Plasmacytoid Dendritic Cell Neoplasm (BPDCN)

BPDCN is a rare and clinically aggressive hematologic tumor derived from cells of immature PDC differentiation (199). The clinical course of BPDCN shows progressive systemic expansion, partially attributed to the local production of chemokine ligands of CKR expressed by the tumor cells (CXCR3, CXCR4, CCR6, CCR7) (200). Beyond expression data, no clues are available on the pathogenic roles of CCR7 in BPDCN.

#### 3.3.4 Langerhans Cell Histiocytosis (LCH)

In LCH pathological CD207^+^ DC show constitutively activated MAPK pathway signaling. In *in vivo* and *in vitro* models, the B-raf V600E activating mutation impaired the Raf/ERK-mediated CCR7-induced migration of DC (201). This in turn caused their retention in the tissue lesions and, by promoting expression of BCL2-like protein 1 (BCL2L1), this resulted in enhanced resistance to apoptosis.

#### 3.3.5 Myeloproliferative Disorders

Chronic myeloid leukemia (CML) is a clonal disease characterized by premature release of aberrant cells from the BM alongside their substantial accumulation in PB, spleen, and BM (202). In CML, the presence of the Philadelphia chromosome and its oncogenic product, the fusion oncoprotein BCR/ABL, is directly linked to multiple pathways involved in cell survival, growth promotion, and disease progression (203, 204). Similarly to LCH, an impaired adhesion and motility towards CCR7 was first reported for CML cells (14, 205) though this effect remains controversial since more recent reports showed *in vitro* and *in vivo* how a positive activation loop between BCR-ABL and the signal-transducing adaptor protein-2 (STAP-2) led to enhanced ERK signaling resulting in overexpression of CCR7, LN enlargement, and hepatosplenomegaly (203, 204). Whether these contradictory outcomes are a result of differential *in vitro versus in vivo* settings, or a consequence of artifacts associated to the use of cell lines *versus* primary tumor cells, needs clarification.

## 4 Pathophysiological Role of CCR7 in Hematologic Malignancies

Chemokines obey Stephen Paget’s ‘seed and soil’ paradigm, proposing that the microenvironments of different organs are different from each other, and that certain tumor cells have specific attraction to the milieu of specific organs (206). As reviewed above, CCR7 is a single receptor driving immune cells into LN, and for this reason this receptor assumes a central role in the pathogenesis of many leukemia and lymphomas, which very often express CCR7 due to their lymphoid or myeloid origin (**Table 1**).

**Table 1:** Summary of blood cancers with reported CCR7 expression studies (following 2016 WHO classification of blood neoplasms) (207).

				CCR7	
				GEP	Protein
**Lymphoid neoplasms**	**Precursor lymphoid neoplasms**	B-ALL and B-lymphoblastic lymphoma		-/+	-/+
		T-ALL and T-lymphoblastic lymphoma		+	+
	**Mature B-cell neoplasms**	Chronic lymphocytic leukemia/small lymphocytic lymphoma		+	+
		Monoclonal B-cell lymphocytosis		na	+
		Splenic marginal zone lymphoma		–	–
		Hairy cell leukemia		–	–
		Lymphoplasmacytic lymphoma/Waldenström macroglobulinemia		na	-/+
		Monoclonal gammopathy of undetermined significance		–	na
		Plasma cell myeloma		–	-/+
		Plasma cell myeloma variants	Smoldering myeloma	–	na
			Non-secretory myeloma	na	–
			Plasma cell leukemia	–	na
		Extranodal marginal zone lymphoma of mucosa-associated lymphoid tissue (MALT lymphoma)		-/+	+
		Nodal marginal zone lymphoma		na	na
		Follicular lymphoma		-/+	-/+
		Primary cutaneous follicle center lymphoma		-/+	na
		Mantle cell lymphoma		+	+
		Diffuse large B-cell lymphoma	GCB type	-/+	-/+
			ABC type	+	+
		T-cell/histiocyte-rich large B-cell lymphoma		-/+	na
		Primary diffuse large B-cell lymphoma of the central nervous system		na	+
		EBV-positive diffuse large B-cell lymphoma		-/+	na
		Primary effusion lymphoma		+	na
		Burkitt lymphoma		-/+	+
	**Mature T- and NK-cell neoplasms**	T-cell prolymphocytic leukemia		+	+
		T-cell large granular lymphocytic leukemia		na	–
		Chronic lymphoproliferative disorder of NK cells		na	–
		Aggressive NK-cell leukemia		na	–
		Adult T-cell leukemia/lymphoma		+	+
		Extranodal NK-/T-cell lymphoma		na	-/+
		Mycosis fungoides		+	+
		Sézary syndrome		+	+
		Primary cutaneous CD30^+^ T-cell lymphoproliferative disorders	Primary cutaneous anaplastic large cell lymphoma	-/+	na
		Primary cutaneous peripheral T-cell lymphomas, rare subtypes	Pimary cutaneous CD8^+^ aggressive epidermotropic cytotoxic T-cell lymphoma	na	–
		Peripheral T-cell lymphoma, not otherwise specified		-/+	+
		Angioimmunoblastic T-cell lymphoma		-/+	-/+
		Anaplastic large-cell lymphoma	ALK-positive	-/+	-/+
			ALK-negative	+	+
	**Hodgkin lymphoma**	Nodular lymphocyte predominant Hodgkin lymphoma		-/+	–
		Classical Hodgkin lymphoma		+	+
**Histiocytic and DC neoplasms**	**Tumors derived from Langerhans cells**	Langerhans cell histiocytosis		–	–
**Myeloid neoplasms**	**Myelodysplastic syndromes**			na	na
	**Acute myeloid leukemia and related neoplasms**			-/+	-/+
	**Blastic plasmacytoid dendritic cell neoplasm**			na	+
	**Myeloproliferative neoplasms**	Chronic myeloid leukemia		–	–

ABC, activated B-cell like; ALK, anaplastic-lymphoma kinase; DC, dendritic cells; EBV, Epstein-Barr virus; GCB, germinal center B-cell like; GEP, gene expression profile; na, not available.

**Figure 1:**
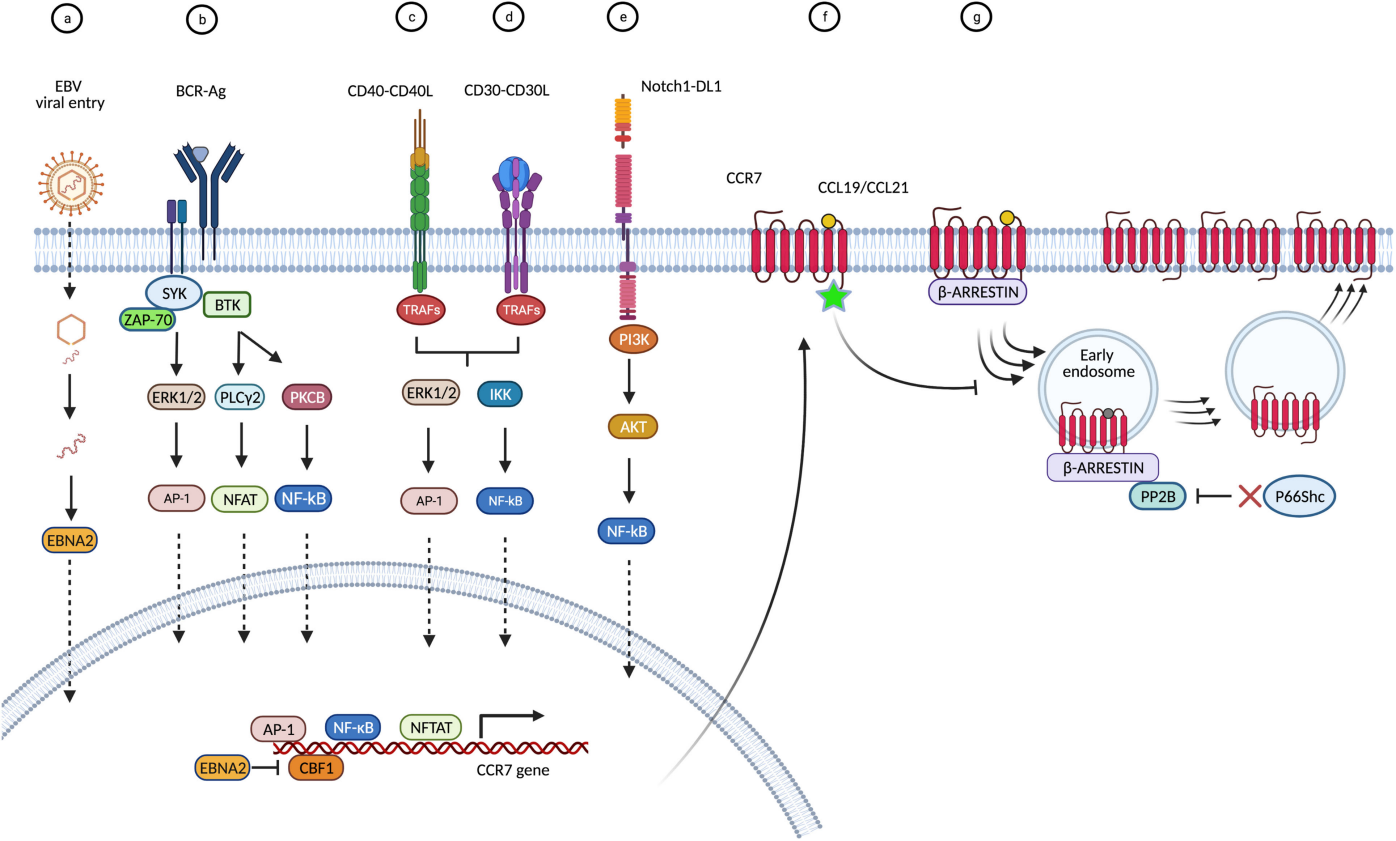
Causes and modes that underlie CCR7 overexpression in blood cancers. Overview of proposed signaling cascades with reported data. **(A)** CCR7 upregulation may be promoted by EBV. In BL and DLBCL cells, following viral endocytosis, the virion and packaged proteins are released into the cytoplasm. The viral transactivator EBNA-2 binds to and inhibits the *CCR7* gene repressor factor CBF-1 (also known as RBP-Jκ) thus promoting *CCR7* gene transcription (4, 89, 91). **(B)** In B-cell malignancies (CLL, B-ALL) tonic signaling through the BCR activates transcription factors such as NFATC1, NF-κB, and AP-1, which target the *CCR7* gene (42, 54, 56). **(C)** When engaged by CD40L, the receptor CD40 recruits tumor necrosis factor receptor-associated proteins (TRAF) to the membrane, which initiate different signaling pathways leading to activation of NFκB or AP-1 (22, 23, 208, 209). **(D)** In cHL and ALCL, binding of CD30L (CD153) or sCD30 to CD30 can result in trimerization and signal mediation through TRAF proteins to stimulate the NFκB pathway resulting in *CCR7* gene expression (3, 210). In addition, CD30 can signal through MAPK pathways, including ERK1/2 and the nuclear transcription factor AP-1, all leading to enhanced *CCR7* transcription (3, 115, 117). **(E)** In T-ALL cells, release of intracellular Notch1 (ICN1) from membrane-tethered heterodimeric Notch1 protein upregulates PI3K/mTORC2/NF-κB pathways and activation of the *CCR7* gene (25, 133). In CLL cells, activating mutations in Notch1 intracellular domain favor the downmodulation of DUSP22 phosphatase thus facilitating the accumulation of activated STAT3 which mediates *CCR7* gene transcription (51). **(F)** Mutations in the C-terminal amino acid Trp355, located in the cytoplasmic region of CCR7, impair internalization upon ligand stimulation resulting in increased expression of the surface receptor in ATLL cells (162, 163). **(G)** Dysregulation of the endocytic machinery of CCR7, e.g. in CLL, impacts receptor turn-over and increases CCR7 membrane expression. Deficiency of the cytoplasmic p66Shc protein causes enhanced activity of the PP2B/calcineurin phosphatase on the endosomal CCR7 pool, which enhances its recycling back to the plasma membrane (43, 46).

In lymphoid malignancies, the role of CCR7 in hallmark deregulations of cancer such as enhanced migration or death resistance, can be associated to functional differences between CCR7-expressing normal and malignant cells. In some cases, gain-of-function in CCR7 is a consequence of an upregulated transcription and/or protein translation (**Figure 1**). For example, tonic signaling through the BCR or CD40 activates transcription factors such as NFATC1, NF-κB, and AP-1, which target the CCR7 gene, a mechanism found in CLL and B-ALL (22, 23, 42, 54–56, 208, 211). Similarly, CD30 down-stream signaling seems to increase CCR7 gene transcription in cHL and ALCL likely through NF-κB and AP-1 (3, 115, 117). Moreover, constitutive activation of the Notch1 oncoprotein increases CCR7 expression in T-ALL through the mTORC2/NF-κB cascade (25, 133), or in CLL through down-modulation of the DUSP22 phosphatase levels and the subsequent increase in STAT3 activation (51). In other instances, CCR7 up-regulation is promoted by a viral machinery that suppresses CCR7 gene repressor factors like CBF-1. This is described for the viral transactivator EBNA-2 in BL and DLBCL (89, 91) and it could be hypothesized that a similar mechanism governs HTLV-1-induced transformation in ATLL. Notably, downregulation of CCR7 expression and reduction of associated chemotaxis during viral infections, have been reported (11, 212). The EBV (213), the murine lymphocytic choriomeningitis virus (LCMV) (214), the human immunodeficiency virus type 1(HIV-1) (215), or the influenza virus (216) are examples of CCR7-downmodulating viruses. In other cases, e.g. during HIV-1 infection, primary CD4^+^ T-cells showed and enhancement of CCR7-mediated motility, leading to efficient propagation of HIV-1 (217, 218). Based on this, one might be tempted to associate these changes of the CKR expression profile to particular needs of each virus’ cycle. Therefore, discrepancies between outcomes in CCR7 expression after viral infections might be also a consequence of distinct cell-to-cell aspects such as the time elapsed after cell infection, the cell development stage at which the infection takes place, or baseline CCR7 expression by host cell. For example, the impact of *in vitro* EBV infection on CCR7 expression was very different between tonsillar or PB B-cells, being milder (if at all) in the last cell type (213). Moreover, the presence of additional tumorigeneic events in the infected tumor cells may synergize with the viral machinery to induce CCR7 gene expression (219, 220). In agreement, expression of CKR and chemokines in immortalized cell lines differs from that of EBV-infected PB B-cells (89, 213) and different growth requirements, such as the oestrogens, are known to positively regulate viral factors like EBNA2, which subsequently activate CCR7 gene expression (4, 213).

Finally, CCR7 up-regulation may be also promoted by mutations in the C-terminal cytoplasmic region of CCR7 or dysregulation of its endocytic machinery both affect receptor turn-over. For example, in ATLL or CLL cells impaired internalization upon ligand stimulation results in increased surface receptor expression (43, 46, 162, 163). Whatever the underlying reasons for the upregulation of CCR7, in the majority of diseases that are reviewed here, all these events lead to increased numbers of functional receptors at the surface of the tumor cells, which endows them with an increased migratory capacity (13, 20, 35, 40, 46, 99, 115, 173). CCR7-mediated migratory abilities can be selectively potentiated in leukemia/lymphoma cells (as opposed to their normal counterparts) by pre-exposure or co-incubation with other homeostatic chemokines like CXCL12 or CXCL13, as demonstrated in MCL, BL, or SS (78, 90, 183). Although in many entities the molecular mechanisms of CCR7 upregulation remain unknown, it is consistently found across various diseases, e.g. B-ALL, T-ALL, or CLL. Therein, its overexpression is associated with the presence of adverse prognostic factors, e.g. ZAP-70, which seem to directly cooperate with CCR7 towards facilitation of homing to survival niches such as LN or CNS (24, 38, 47). In fact, in CLL ZAP-70 has been shown to govern integrin activation upon CCR7 stimulation, in a G-protein independent fashion and through oligomerization of four CCR7 molecules (58, 63). Nevertheless, some associations of CCR7 expression with markers of disease subsets, hence aggressiveness or outcome, might be of indirect nature and just represent indicators of different inherent cellular programs (e.g. higher migratory potential), as suggested for the histogenetic subsets of CLL with unmutated *IGHV* and/or with trisomy 12 that show higher responsiveness to CCR7 ligands (35, 36, 38, 46, 47, 66).

**Figure 2:**
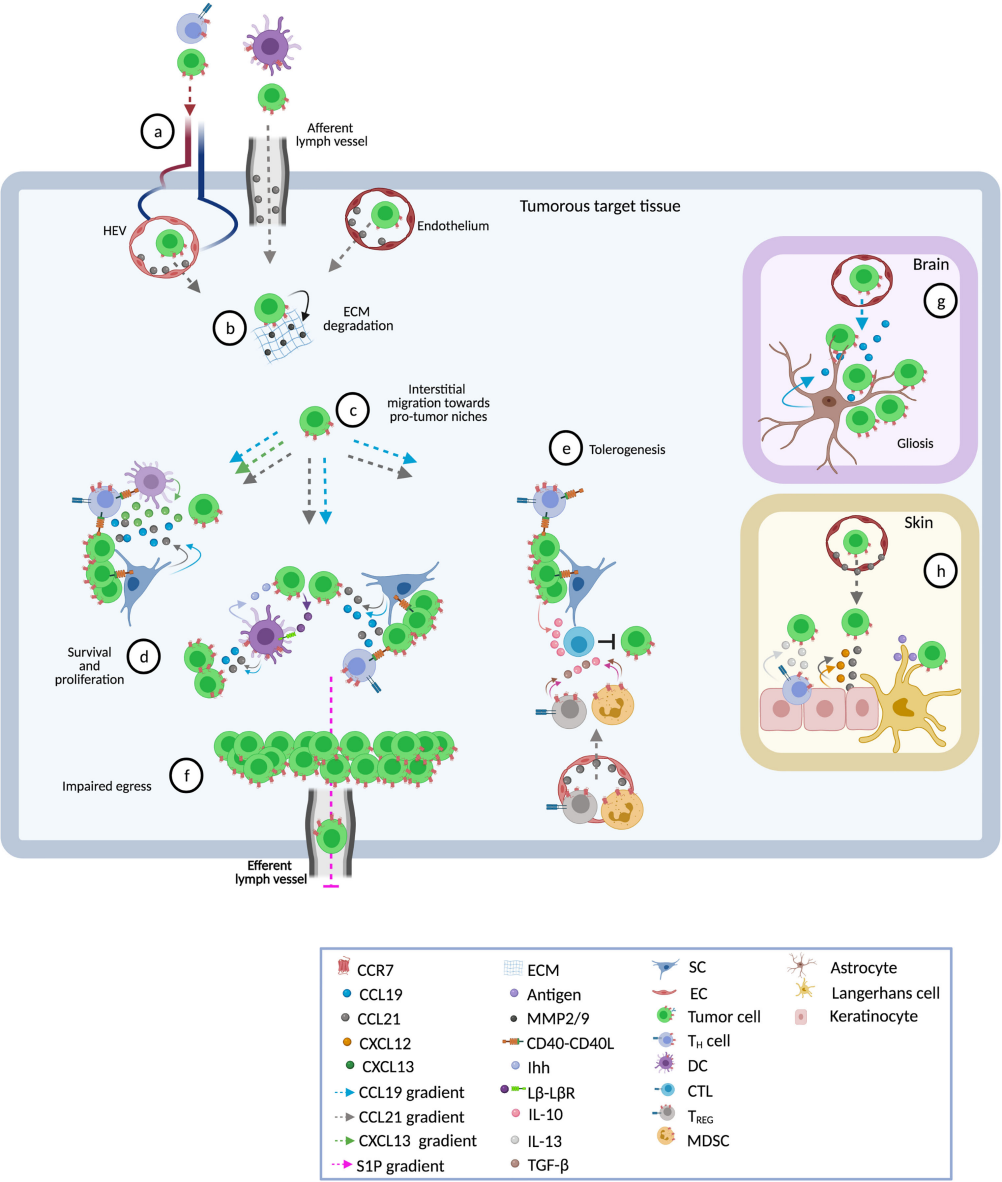
Pathophysiological roles of CCR7 in blood cancers. Shown are different tissues and ways in which the receptor contributes to disease progression. **(A)** Following CCL21 gradients, CCR7-expressing tumor and accessory cells enter into target tissues through high endothelial venules (HEV, in LN and other SLO), afferent lymphatic vessels (in LN), or endothelial cells (BM, spleen, skin, CNS) (8–10, 24, 25, 104, 130, 134). CCL21 on the surface of endothelial cells (EC) activates α4β1 and αLβ2 integrins thus facilitating transmigration (35, 58, 64, 130, 157). **(B)** Subsequently, activated CCR7 promotes invasive phenotypes that secrete metalloproteinases 2 and 9 (MMP-2/9) and promotes extracellular matrix (ECM) degradation (64, 130, 183, 184). **(C)** Within the tissue, CCR7 drives interstitial migration of tumor cells to distinct sites such as T-zones or B-zones in spleen and LN (9), or to lymphoid-like (tertiary) structures in skin or CNS (104, 169). Some tumor cells have inherent abilities to migrate towards CCL19/CCL21 gradients whereas others need cooperative signaling by other chemokines (e.g. CXCL12 or CXCL13) or a previous stimulus, such as exposure to CD40L, to initiate this process (20, 22, 23, 78, 90, 183, 209). **(D)** CCR7-driven interstitial migration assists tumor cells in their right positioning adjacent to accessory cells such as CD40L^+^CD4^+^ T_H_ cells, DC, and stromal cells (SC) which foster growth and resistance to spontaneous or drug-induced cell death (9, 68). Cross-talk with accessory cells induces the release of CCL19 and CCL21 (9, 25, 134) directly promoting survival and proliferation of tumor cells *via* MAP-kinase and PI3K signaling pathways (9, 40, 70, 223). CCL19 can also act in cooperation with CXCL13 to confer resistance to TNF-α-mediated apoptosis *via* up-regulation of PEG10 (26, 27). Other pro-tumor factors delivered by accessory cells include the Indian hedgehog (Ihh) secreted by fibroblastic reticular cells (FRC) and CD40 stimulation through CD40 ligand-expressing CD4^+^ T_H_ cells (9). In turn, malignant cells secrete factors that stimulate and protect accessory cells. One of these factors is lymphotoxin through which tumor cells stimulate lymphotoxin-β-receptor (LβR) expressing FRC (9). Finally, in these niches enhanced production of CCR7 ligands establishes a self-enhancing loop that recruits more tumor and accessory CCR7-expressing cells favoring the continuation of pro-tumor microenvironments (9, 25, 134, 161, 169, 221). **(E)** CCR7 ligands may attract CCR7^+^ immunosuppressive cells such as T_REG_ and myeloid-derived suppressor cells (MDSC) (224, 225). These suppressor cells inhibit anti-tumor effector cells (e.g. CTL) through cell-cell interactions or by creating a tolerant milieu enriched in suppressor cytokines like IL-10 and tumor growth factor beta (TGF-β) (22, 28, 225, 226). Similarly, CCL19 and CXCL13 may synergistically regulate CD40-CD40L cross-talk between cancer cells and CD8^+^ T-cells leading to a PEG10-mediated enhanced production of IL-10 in CD40-activated tumor cells that inhibits tumor-specific CTL (28). Together, these CCR7-induced mechanisms facilitate permissive milieus within tumor target tissues. **(F)** CCR7 also prolongs the time of residence of CCR7-expressing cells in lymphoid tissues (i.e. LN) thus favoring proliferative cycles and providing niches of escape from systemic therapies. In the steady state, internalization of CCR7 activates the transcription and surface expression of S1P1, facilitating the egress of lymph-node-homed immune cells through efferent lymphatic vessels (71). Tumor-associated overexpression of CCR7 impairs S1P1 upregulation thus retaining tumor cells within the LNs, reducing the egress and causing lymphadenopathy (43, 46, 72). (**G)** In CNS, astrocyte-derived CCL19 attracts tumor cells and enhances their parenchymal retention thus contributing to gliosis (24, 25, 104). Inside cerebral or spleen parenchyma, cross-talk between stromal cells and leukemic cells mediates the production of higher levels of tissue CCL19 (25, 134) facilitating the infiltration of tumor cells. **(H)** In the skin, CCL21 in the cytoplasm of epidermal keratinocytes and to be diffusely distributed in the dermal extracellular matrix may lead tumor cells to milieus enriched in growth factors such as CXCL12, IL-13 or antigens able to entertain chronic T-cell receptor stimulation (169).

Generally, in most blood cancers, CCR7 expression correlates with nodal or spleen involvement. In the conditions of B-ALL, MCL, T-ALL, or T-PLL it is also associated with infiltration of the CNS and in CTCL it correlates with the degree of epidermotropism (21, 24, 25, 130, 134, 161, 169, 221, 222). Therefore, it is consistently proposed that overexpression of CCR7 confers an invasive phenotype that contributes to lymphatic and hematogenous spread and promotes homing into target tissues (**Figure 2**). This CCR7^+^ transmigrating phenotype is further characterized by activation of α4β1 and αLβ2 integrins that facilitate adhesion of malignant cells to HEV or stromal proteins, and that promote the secretion of matrix metalloproteases MMP-2 and/or MMP-9, which degrade extracellular matrix (35, 58, 64, 130, 157, 183, 184). Both events result in transmigration of tumor cells into protective niches at which, particularly in T-zones, CCR7 contributes to disease progression in four major ways:

**1)** In ‘homing’ tumor cells, CCR7 drives interstitial migration within the tissue and assists in optimal positioning, e.g. adjacent to accessory cells such as CD40L^+^CD4^+^ T_H_ cells, DC, and stromal cells, which foster growth and resistance to spontaneous or drug-induced cell death. As described, this positioning can be controlled by local factors including gradients of CCR7 ligands (9, 69, 104, 121), CD40-CD40L interactions (9, 68), BCR/ZAP-70 signal transduction (38, 42), or canonical WNT signaling (119).

**2)** Crosstalk with accessory cells can induce the release of high levels of the chemokines CCL19 and CCL21, which engage and activate CCR7 to provide pro-survival signals (9, 25, 134). In addition, stromal cells can produce CCR7 ligands in a constitutive manner (35, 46, 121, 161, 169, 223). Whatever the source of CCR7 ligands, they promote survival, e.g. by acting as trophic factors that induce MAPK and PI3K-AKT signaling (9, 25, 40, 70, 134, 223). They can also act in cooperation with CXCL13, which contributes to resistance to TNFα-mediated apoptosis preferentially in malignant over normal B-cells, *via* upregulation of *PEG10* and stabilization of caspases-3 and -8 (26, 27). In some entities, e.g. MCL, T-PLL, or SS, CCR7 ligands have also shown to trigger proliferation (21, 130, 169).

**3)** Besides promoting influx, CCR7 (together with S1P1) also contributes to LN enlargement by regulating egress from lymphoid tissues, hence accumulation. It prolongs the time of residence of CCR7-expressing cells in lymphoid tissues, by that favoring proliferative cycles and providing niches of escape from systemic therapies. In CLL, the characteristic high expression of CCR7 can be attributed to abnormalities in the surface membrane recycling machinery as a consequence of abnormally low production of the cytoplasmatic p66Shc protein (43, 46). Deficiency of the cytoplasmic p66Shc protein causes enhanced activity of the PP2B/calcineurin phosphatase on the endosomal CCR7 pool, which enhances its recycling back to the plasma membrane. Likewise, ATLL cells carry mutations in the C-terminal cytoplasmic domain of CCR7 that impairs proper recycling (162, 163).

**4)** CCR7 participates in the creation of permissive tumor microenvironments within tumorous target tissues (SLOs, CNS, or skin). As part of the involved tissue interactions between tumor cells and stromal or other accessory cells, an increased production of CCR7 ligands is stimulated (9, 25, 134). For example, cooperative CCR7/WNT signaling is needed for secretion of VGEF-A by cHL cells, which leads to *de novo* generation of vascular endothelium which, in turn, presents CCR7 ligands that direct movement of cHL towards vascular niches (119). Similarly, BL cells can secrete lymphotoxin through which they stimulate lymphotoxin-β-receptor expressing gp38^+^ FRC that secrete the survival factors Ihh and CCL21 (9). These patterns and higher amounts of chemokines establish a feed-forward loop that not only recruits additional CCR7-expressing malignant cells, but also further supportive bystander cells (9, 25, 105, 134, 161, 221). Moreover, in B-ALL CCR7 ligands potentiate secretion of immunosuppressive IL-10 by tumor cells leading to impaired specific anti-tumor CTL responses (28). CCR7 can also recruit regulatory cells that hamper anti-tumor immunity. For example, an increase of functional T_REG_ has been established for patients with blood cancers (226). Expansion of T_REG_ is needed to generate and sustain a tolerogenic TME (227). Indeed, higher numbers of T_REG_ associate with progressive disease in cHL, CLL, MGUS, MM, or DLBCL (228–232).

## 5 Biased Signaling of CCR7 Ligands in Blood Cancers

In the field of GPCRs, knowledge on the diversity in signaling pathways has promoted the concept of “biased signaling”, which involves a context-specific preference for one intracellular signaling pathway over another (233). This concept can be considered as either receptor bias (the same ligand has different actions through different receptors), ligand bias (more than one or naturally modified ligands act on the same receptor and induce different outcomes), or tissue bias (the cellular effect depends on the tissue/cell type) (234). How biased signaling enables different downstream pathways that eventually will determine the overall outcome of CCR7 engagement in different immune cell types has been recently, and deeply, reviewed by Hauser et al. (11). The role(s) of biased signaling of CCR7 in the pathophysiology of blood cancers is not clear, although a scarce number of studies comparing CCR7 activation in healthy *versus* malignant cells indicate that a differential regulation is plausible. For example, in CLL we demonstrated that PI3K and ROCK, but not MAPK, were involved in migration of CLL cells toward CCL19 and CCL21, whereas normal B-cells relied more on PI3K, ROCK, and p38-SAPK pathways (40). Moreover, while CLL cells showed an enhanced, similar migratory response to both CCR7 ligands, normal B-cells showed a moderate response, and preferentially towards CCL21 (13, 35, 40, 46). Likewise, CLL and normal B-cells showed a different signaling to migrate through the endothelium (35, 235). Finally, it is worth mentioning that in the last years CCR7 has been shown to form heterodimers with CXCR4 (217). This process affects CCR7 signaling and might explain a poorly understood, but important, mechanism of chemokine biology that allows synergistic and/or inhibitory outputs produced by simultaneous activation or inhibition of multiple CKRs. Interestingly, CCR7/CXCR4 dimers may enhance tumor B-cell homing to LN by potentiating the TEM upon simultaneous exposure to CXCL12, CCL19, and CCL21 (90) while in healthy, mature B-cells CCR7 acts as a selective allosteric modulator that inactivates CXCR4 thus impairing retention in the BM (236). Together, these findings support the existence of a functional and phenotypic diversity as a result of a biased signaling of CCR7 in homeostasis and blood cancers. Nonetheless, other studies show identical mechanisms in both healthy and neoplastic cells. For example, CCL19-specific translation of S1P1 is mediated by ERK-5/Krüppel-like factor-2 in the HuT78 SS cell line and healthy primary T-cells (237). Therefore, additional comparative studies addressing biased signaling in both healthy and tumor tissues are mandatory to better understand the pathophysiological roles of these regulatory mechanisms in CCR7 and other CKRs.

## 6 CCR7 as a Therapeutic Target in Blood Cancers

The circumstantial and direct evidence presented in this review suggests that the tumor-associated CCR7-ligand interaction is an actionable vulnerability. At CCR7-ligand permissive sites, e.g. LN or brain, malignant cells evade spontaneous or drug-induced apoptosis as well as escape immune cell control or proliferate, all in a CCR7-mediated manner. Therefore, interfering in CCR7-signalling promises to be of therapeutic potential in many CCR7 expressing and CCR7 promoted blood cancers. Nonetheless, targeting CCR7 in cancer has the potential downsite of activating and/or potentiating alternative pathways that would eventually allow homing of tumor cells to protective niches. For instance, to adhere to HEVs of peripheral and mesenteric LNs T-cells rely on CCR7 and partially on CXCL12/CXCR4 whereas normal B-cells can exploit the CXCR4 and CXCL13/CXCR5 axes to induce integrin-mediated arrest on HEVs and homing to the LN (238–243). To avoid this scenario, it is desirable that anti-CCR7 drugs feature a double MOA including both, neutralization of the target and tumoricidal capacities. In this regard, we and others have demonstrated that approaches based on we and others have demonstrated that approaches based on blocking (non-activating) monoclonal antibodies (mAbs) that target CCR7 or its ligands, are highly effective in *in vitro* and *in vivo* preclinical models, including B-ALL (24), CLL (37, 40, 45), MCL (21), T-ALL (24, 25), or T-PLL (130). In these studies, such anti-CCR7 therapies reduced tumor cell migration and infiltration into CCR7-specific environments and additionally impaired survival/proliferation. Overall, the combined neutralizing and killing activities of anti-CCR7 mAbs led to retarded tumor implantation, reduced tumor burden, and significantly extended host survival in *in vivo* models.

Taken together, there is ample data on target expression and mechanistic rationales as well as sufficient proof of principle and feasibility data that strongly encourage the therapeutic application of anti-CCR7 therapies in blood cancers. Consequently, first clinical-grade anti-CCR7 antibodies have been developed during the last years. Novartis is enrolling patients into a phase-I trial with JBH492 an antibody-drug conjugate (ADC) targeting CCR7 (NCT042140704). Moreover, Catapult Therapeutics presented first pre-clinical results of an antagonist mAb called CAP-100 that will be evaluated in first-in-human clinical trials in 2021 (NCT04704323) (244). In preclinical settings, both compounds have shown to be highly effective as a single agent and at least CAP-100 revealed the potential for combinations with current standard-of-care drugs (245). Owing to their particular MOA, antagonisticanti-CCR7 mAbs may be likely combined with other standard-of-care drugs to obtain additive or synergistic effects while reducing the likelihood of treatment resistances. For example, by blocking ligand-receptor interactions, anti-CCR7 therapies may displace tumor cells out of protective niches, forcing them to accumulate in blood where they may become more accessible to other cytotoxic drugs such antibodies against established targets (e.g. CD20, CD30, CCR4, etc), or chemotherapeutics (e.g. fludarabine), or small molecule inhibitors (e.g. BCL2 inhibitors). Along with the BTK inhibitor ibrutinib or with the PI3Kδ inhibitor idelalisib, anti-CCR7 mAbs would additively or synergistically target CCR7-mediated adhesion to lymphoid stroma or endothelium, thus favoring an enhanced cell egress from lymphoid tissues into circulation (44, 46, 48, 246). In fact, we have recently demonstrated that CCR7 expression and functionality is not impaired during ibrutinib treatment in CLL patients and that the anti-CCR7 CAP-100 and ibrutinib show complementary activities (245). Moreover, while the antibody would block recirculation and loops of LN homing, ibrutinib would also interfere with CXCR4- and CXCR5-mediated signaling and with the production of chemokines (CXCL12, CXCL13, CCL19) by myeloid stroma cells (44, 247), thus acting against potential redundant chemotactic pathways. Finally, it is worth mentioning that immune checkpoint blockade and CAR-T-cells have revolutionized the field of cancer therapy during the last decades. Whether anti-CCR7 therapy may complement such treatments is uncertain since for avoiding negative interactions it seems necessary that therapeutic T-cells to express an effector or effector memory CCR7-negative phenotype.

Given the various roles in tumor development and progression, adhesion molecules are promising targets to block the access of tumor cells to tumor-permissive niches like the LN (248, 249). For instance, LFA-1 and VLA-4 are involved in the development of hematological malignancies and tumor cells require their expression to migrate into lymphoid tissues (250, 251). Therefore, it is plausible to speculate that targeting these leukocyte adhesion molecules might be an alternative way to target the CCR7 axis. Like anti-CCR7 assets do, targeting cell adhesion exerts direct effects to the tumor cell (e.g. reduction of motility, invasiveness, and proliferation) that may impair homing to SLO (252, 253). In addition, cell adhesion molecules are common downstream players activated by several CKRs, including CCR7 (254), hence, their inhibition would potentially inhibit CCR7 along with several other receptors, thus overcoming the redundancy of CKRs (255). Yanguas et al. showed in murine melanoma models that an increased number of intra-tumorally injected tumor-specific T-cells migrated into the draining LN when treated with anti-ICAM-1 or anti-LFA-1 mAbs (256). This indicates that specific approaches, such as anti-CCR7 mAbs, are needed to block LN homing. Moreover, since integrins play diverse roles in immunity and anti-tumor responses, targeting the function of these molecules *in vivo* may be a difficult task in cancer therapy (254, 257, 258). Accordingly, multiple clinical trials that involve the targeting of αV or β1 integrins have shown disappointing results with low therapeutic efficacies (249), while anti-LFA-1 strategies have been associated with an increased risk of malignancies, infections, and rare, but severe, systemic adverse events such as immune-mediated thrombocytopenia and hemolytic anemia (259, 260). To overcome these deleterious effects, novel approaches aiming to target the integrin and/or ligands on tumor cells or tumor vessels, but not in immune cells, are needed. Bispecific antibodies simultaneously directed against LFA-1 and a tumor specific antigen may contribute to specifically block LFA-1-mediated tumor cell adhesion without affecting immune responses, as shown in mice (261). Since targeting CCR7 shares both overlapping and differentiating MOAs with such therapies that target adhesion, combining both of these strategies could provide clinical benefits and needs to be further investigated.

## 7 Safety of Novel Therapies Targeting CCR7 in Blood Cancers

Currently, two clinical trials aim to validate anti-CCR7 approaches in hematologic diseases with an urgent need for more rationally based and efficient therapies. These studies will also allow us to learn the real risks that are associated with blocking CCR7 and/or depleting CCR7-expressing immune cell subsets as this receptor is critical for activation steps in the adaptive immune system and for the homeostasis of T_REG_, which limit self-reactive events and autoimmunity (262). In one hand, several mouse models revealed that deficiency of CCR7 signaling was not a life-threatening condition, as it was associated with a moderate impact on immunity by retarded, but preserved, T-cell and B-cell responses (8, 240, 263–265), especially against infections with replicating antigens (266–269). However, in different vaccinations approaches (e.g. HIV, HSV, or HCV) adjuvant CCL19 was relevant for augmenting the trafficking of T-cells and DC (270–272). Therefore, anti-CCR7 therapy may reduce priming of antigen-specific T-cells and the production of Abs in a virus-dependent manner. From pre-clinical models, we know that the use of anti-CCR7 mAbs selectively inhibited and/or depleted tumor cells while sparing healthy counterparts (37, 45, 244, 273, 274). Notably, CD4^+^ T_N_ and T_CM_ cells were preferentially impacted while other CCR7-expressing subsets such as DC or B-cells were not. Lower target density in non-tumor cells or lower affinity of these antibodies for CCR7 expressed in these cell types could explain these observations which also suggests that anti-CCR7 therapy might impair new immunization processes dependent on T_N_ cells, but not memory effector responses against infections (273–275). In this regard, CCR7-negative T_EFF_ and T_EM_ rather than CCR7-expressing T_N_ or T_CM_ are necessary for effective anti-tumour responses (276). Moreover, naïve tumor-specific CD8^+^ T-cells, which seem less susceptible to anti-CCR7 therapy (244, 273, 274), can also become activated and gain effector-cell phenotypes directly at the tumor site, suggesting that cross-presenting DC are also able to prime CD8^+^ T-cells within the tumor (277). These results indicate that DC migration into LN may not even be completely necessary for DC-mediated anti-tumor responses.

Targeting CCR7 could also affect B-cell homing during antigen-dependent and independent B-cell differentiation; however, CCR7-deficient mice show splenic B-cell responses upon bacterial challenge (240). In addition, normal B-cells are less dependent on CCR7 than leukemic cells for the arrest on HEVs and for homing (71, 238, 278) while BM B-cell precursors and plasma cells lack CCR7 (13), suggesting that CCR7 therapy would not suppress B-cell lymphopoiesis nor immunoglobulin secretory function (8, 240).

Related to unwanted autoimmune side-effects, lack of CCR7 signaling in T_REG_ hampered central and peripheral tolerance (224, 279–282) and led to generalized multi-organ autoimmunity. Whether anti-CCR7 therapies will resemble phenotypes in CCR7-deficient animals will remain unknown until first evidence in patients becomes available. Until then, clinical results with the therapeutic antibody mogamulizumab (which removes CCR4^+^ T-cell subsets, including T_REG_) (283) allow us to speculate that anti-CCR7 can be safe and well-tolerated. In line with this, anti-CCR7 therapy in pre-clinical syngeneic mouse models of cancer, autoimmunity, GVHD, or inflammation did not uncover treatment-associated side effects (225, 273, 275) and CAP-100 toxicology studies in NHP did not reveal overt toxicities or autoimmune disease, all indicating superior tolerability of this novel therapy (244). In the coming months, first data in patients receiving a chronic administration of an anti-CCR7 mAb will be available and, hopefully, results from clinical studies will shed light into the safety and utility of targeting CCR7, and more importantly, will validate anti-CCR7 approaches in hematologic diseases with an urgent need for more rationally based and efficient therapies.

## 8 Conclusions

Classically, the pathogenic role of CCR7 as a cancer-associated receptor in hematology has been attributed to its unique ability to drive tumor cells into the LN and other SLO. Accordingly, CCR7 expression has been strongly linked to bulky disease in these lymphoid tissues. Nonetheless, this canonical (and somehow narrow) view of CCR7 as a migratory receptor is changing thanks to recent evidence that supports additional pathogenic functions of CCR7. Beyond cancer cell lymphotropism, we have disclosed that CCR7 expression is also associated to neurotropism and epidermotropism, to interstitial migration within tumor tissues, to juxta-positioning to accessory cells, and to cell survival and proliferation. Moreover, CCR7 also guides different accessory cell types that are needed to create and preserve pro-tumor niches and to protect cancer cells from spontaneous or drug-induced apoptosis. Likewise, immunosuppressive cells take advantage of CCR7 to locate themselves close to innate or adaptative anti-tumor immune cells, thus facilitating their tolerogenic or their inhibitory participation in the TME.

However, our knowledge on CCR7 biology in blood cancers is still scant and additional efforts are needed to solve relevant questions such as around the major mechanisms regulating CCR7 (over)expression, how CCR7 contributes to tumor growth during the first tumorigeneic events, or what the exact contributions of CCR7 in accessory cells in early cancer events or during different stages of target tissue colonization are.

CCR7 is currently postulated as a potential therapeutic target for some blood cancers and novel antibody(conjugate)-based strategies targeting CCR7 are being evaluated in early-phase clinical trials. It is also tempting to speculate that modulation of CCR7 expression and signaling in therapeutic lymphocytes might allow manipulation of the performance (e.g. migratory potential, longevity) of T- or NK-cells carrying chimeric-antigen receptors. If such direct or indirect modulation of tumor-cell or milieu-derived CCR7-signaling stands out as a promising approach it is likely that in the coming years an extensive collection of novel evidence will help to better understand its biology and to refine CCR7-based translational applications.

## Author Contributions

CC-M conceived the manuscript, wrote the manuscript, and conceived and created the figures and tables. FT co-wrote the manuscript. MH co-wrote the manuscript, conceived and edited tables and figures. All authors contributed to the article and approved the submitted version.

## Conflict of Interest

CC-M is an employee of Catapult Therapeutics and of Immunological and Medical Products (IMMED S.L.), and a shareholder in this last company. FT declares that he is CEO and a shareholder in the same companies.

The remaining author declares that the research was conducted in the absence of any commercial or financial relationships that could be construed as a potential conflict of interest.

## Publisher’s Note

All claims expressed in this article are solely those of the authors and do not necessarily represent those of their affiliated organizations, or those of the publisher, the editors and the reviewers. Any product that may be evaluated in this article, or claim that may be made by its manufacturer, is not guaranteed or endorsed by the publisher.
